# National post-market surveillance assessment of veterinary medicines in Korea during the past decade

**DOI:** 10.1186/s12917-017-1054-z

**Published:** 2017-05-22

**Authors:** JeongWoo Kang, Hae-chul Park, Yang ho Jang, Md Akil Hossain, Kyunghun Jeong, Mi young Jeong, Seon-Jong Yun, Sung-won Park, Dae gyun Kim, Kwang-jick Lee

**Affiliations:** Veterinary drugs & Biologics Division, Animal and Plant Quarantine Agency (QIA), 177, Hyeoksin 8-ro, Gimcheon-si, Gyeongsangbuk-do 39660 Republic of Korea

**Keywords:** Veterinary medicine, Quality control, Post-market surveillance assay

## Abstract

**Background:**

Veterinary medicines have been widely used for the prevention and treatment of diseases, growth promotion, and to promote feeding efficacy in livestock. As the veterinary medicine industry has steadily grown, it is crucial to set up a baseline for the quality of medicine as well as the insufficiency or excessiveness of the active ingredients in drug products to ensure the compliance, safety and efficacy of these medicines. Thus, the 10 years data of post-marketing quality control study was summarized to determine the rate and extent of non-compliance of these medicines and to establish baseline data for future quality control measures of veterinary medicine.

**Results:**

In this study, 1650 drugs for veterinary use were collected per year from each city and province in Korea and analysed for the quantity of active ingredients according to the “national post-market surveillance (NPMS) system” over the past decade. The NPMS assessment was performed using liquid and gas chromatography, titration, UV/Vis spectrophotometry, and bioassays. A total of 358 cases were deemed noncompliant, with the average noncompliance rate for all medicine types being 2.0%. The average noncompliance rates for antibiotics, biologics and other chemical drugs except antibiotics (OCD) were 1.1%, 1.2%, and 3.0%, respectively. The first leading cause for noncompliant products was insufficient quantity of major ingredients (283 cases), and the second leading cause was the existence of excess amount of active ingredients (60 cases). Tylosin, spiramycin, ampicillin, tetracyclines and penicillins were most frequently found to be noncompliant among antibiotics. Among the OCD, the noncompliance was found commonly in vitamin A.

**Conclusion:**

The overall trend presented gradually decreasing violation rates, suggesting that the quality of veterinary medicines has improved. Consistent application of the NPMS assessment and the establishment of the Korea Veterinary Good Manufacturing Practice (KVGMP) will help to maintain the good quality of medicine.

## Background

Veterinary medicines are concerned with the prevention, control, diagnosis, and treatment of diseases that affect the health of companion, domestic, exotic, wildlife, and production animals. They are also used to improve feed efficiency, promote growth of livestock, and prevent the transmission of animal diseases to people. The veterinary medicine industry in Korea has grown following the expansion of livestock industry, and reached a market size of 876 millions USD by 2016. As of 2016, the total number of domestic manufacturers and importers of livestock are 740, consisting of 374 manufacturers, 358 importers, and 8 repair shops of medical devices, while the number of approved products reached a total of 9160 (4000 antibiotics, 2160 biologicals and 3000 other veterinary drugs except antibiotics and biologics [OCD]). In Korea, national product quality control measures for veterinary medicine consists of pre-market government testing system (PMGTS) and the National Post-Market Surveillance (NPMS) assessment on veterinary medicine before and after the distribution. Quality controls of antibiotics and biologics are performed by PMGTS prior to reach the products in open market, ensuring that stringent efficacy and safety requirements are met in accordance with the “Handling Rules of Veterinary Medicinal Products” [1, 2].

Meanwhile, local manufacturing facilities and veterinary medicinal products quality have improved owing to the implementation of Korea Veterinary Good Manufacturing Practice (KVGMP) on 1988–05-18 in accordance with the “KVGMP requirement for quality control” resulting in the gradual decrease of the government testing rejection rate [3, 4]. Accordingly, testing of antibiotics by PMGTS was abolished from 2000 to 11-07, while some biologics received partial exemptions from PMGTS as of 2005–04-09. The latter exemptions are limited to KVGMP-designated manufacturers. For biologics, PMGTS is performed on newly approved products, which become exempt from government testing after 10 or more consecutive lots are found to be acceptable. Following acquisition of exempt status, these products are still subject to spot-check government testing when necessary. Moreover, in the case of new antibiotics, the first 5 manufactured lots are designated as priority collection items and collected for testing [3, 5].

**Fig. 1:**
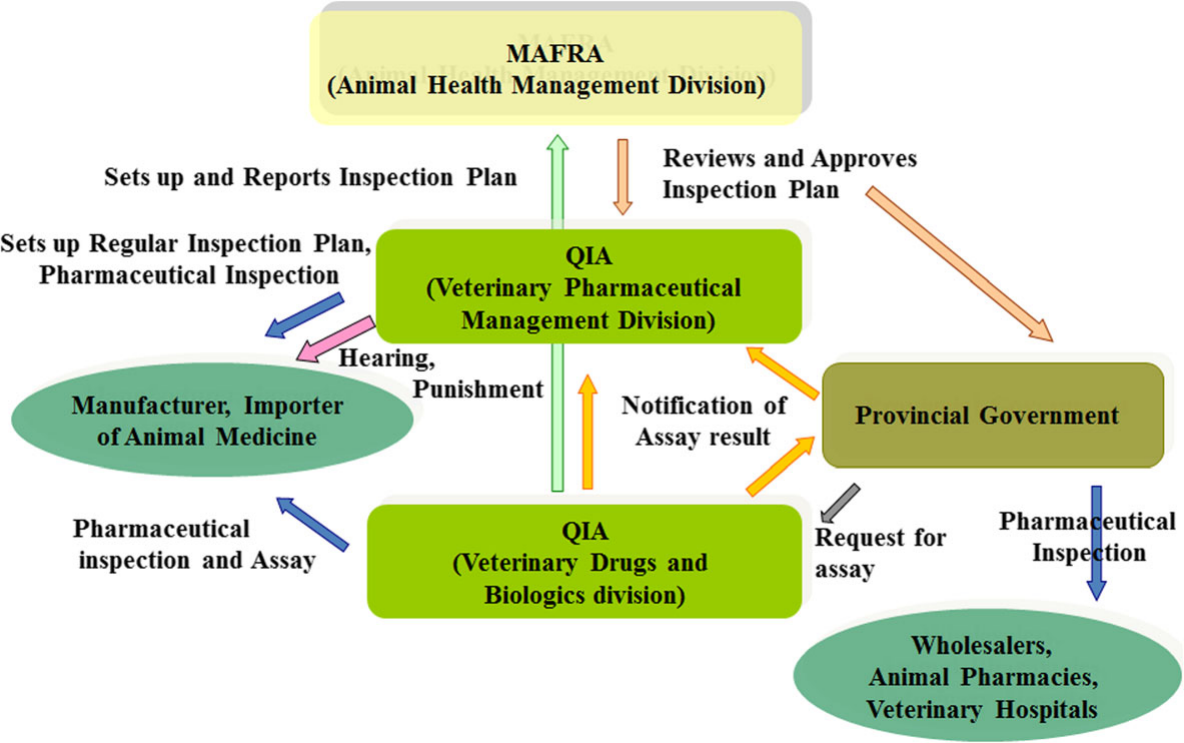
Schematic figure showing Pharmaceutical inspection of veterinary medicines by post-market surveillance assessment. MAFRA: Ministry of Agriculture, Food and Rural Affairs; QIA: Animal and Plant Quarantine Agency

In the case of veterinary medicines currently in distribution, the active ingredient quantities of more than 1650 medicines are tested each year in accordance with “Tips for inspecting a veterinary pharmaceutical affair” [5]. The Korean Ministry of Agriculture, Food, and Rural Affairs (MAFRA) has established collection and testing plans designating the number of products, types of active ingredients, and locations from which sampling should take place (Fig. 1). The Korea Animal and Plant Quarantine Agency (QIA) and each individual city/province collects antibiotics, biologics and OCD currently in distribution from manufacturers, importers, and wholesalers of veterinary medicine, veterinary pharmacies, and veterinary hospitals in accordance with these testing plans. During this process, products are collected, with priority given to products with questionable quality, products with sale prices below the cost of production which disrupt the distribution order, identical products sold at widely disparate prices, and products with high market share. Post-marketing quality control measures are implemented on these products through QIA testing and levying of administrative measures such as suspension of marketing authorization and withdrawal of marketing authorization [1, 5]. The present study was performed to analyse the results of these veterinary medicine collections and tests over the past 10 years, between 2006 and 2016, for the purpose of establishing baseline data for future veterinary medicine quality control measures.

## Methods

### Test sample collection

The tested samples consisted of veterinary medicines stored or distributed by manufacturers, importers, and wholesalers of veterinary medicine, veterinary pharmacies, and veterinary hospitals, and were collected by QIA and local municipalities throughout Korea (9 provinces and 8 cities). In total, 18,213 products were collected and tested from 2006 to 2016. All the drug samples were analysed three times immediately after collecting them, and the results were documented.

### Chemicals and reagents

Reference standards were purchased from Sigma-Aldrich (St. Louis, MO, USA) for all the veterinary drugs. All solvents used in chromatographic analysis were of HPLC grade and were purchased from Honeywell Burdick & Jackson (Ulsan 680–160, South Korea). De-ionized water was purified using a Milli-Q System (Millipore, Bedford, MA 01730, USA). Solvents and reagents used in titrimetric assays were reagent grade and were obtained from Duksan Pure Chemical Co., Ltd. (Ansan-si 425–020, South Korea) and Honeywell Burdick & Jackson (Ulsan 680–160, South Korea).

### Test methods

Collected veterinary medicine samples were tested by the QIA using various certified methods such as the Korean standards of veterinary pharmaceuticals [6], the Korean pharmacopoeia [7], the Standards of national certification assay of the veterinary biological products [8] and other foreign test methods which are summarized in “Compendial analysis method for veterinary medicines, Animal and Plant Quarantine Agency (QIA), South Korea” (Table 1). [9–13] High Performance Liquid Chromatography (HPLC) and microbial assays were used for antibiotics while HPLC, pH, and titration methods were used for other chemical agents. Finally, vaccines, classified as biologics, were tested using microbial and viral assays.

**Table 1:** Outlines of analysis veterinary medicines

Veterinary medicines	Active ingredient	Analytical method (instrumentation)
Antibiotics	Penicillins	HPLC
	Cephalosporins	HPLC
	Tetracyclines	HPLC
	Sulfonamides	HPLC
	Quinolones	HPLC
	Macrolides	HPLC
	Aminoglykosides	HPLC
	Others (avilamycin, colistin, trimethoprim)	HPLC
	Amikacin	Bioassay
OCD	Anthelmintics	HPLC
	Antiprotozoals	HPLC
	Hormones	HPLC
	Paracetamol	HPLC
	Other Antipyretics	Titration
	Vitamins	HPLC
	Nutrition facts	Titration
	Others (tartaric acid, sorbitol)	Titration, pH
	Disinfectants (malic acid, O-Dichlorobenzene, Cresol, Sodium dichloroisocyanurate, Citric acid, Alkylpolyamine ethylglycine, Chlorhexidine digluconate)	HPLC
	Disinfectants (Potassium monopersulphate triple salt, Quaternary ammonium chloride, Alkyldiaminoethylglycine, Hydrogen peroxide, Available chloride, Sodium hypochlorite, Glutaraldehyde, Aldehyde, chlorine dioxide, sodium carbonate, iodine, sodium hydroxide)	Titration
	Probiotics	Bioassay, pH
Biologics	Vaccines	Bioassay, Elisa assay

*OCD* other chemical drugs except antibiotics

## Results

### National post-market surveillance assessment results by year

**Fig. 2:**
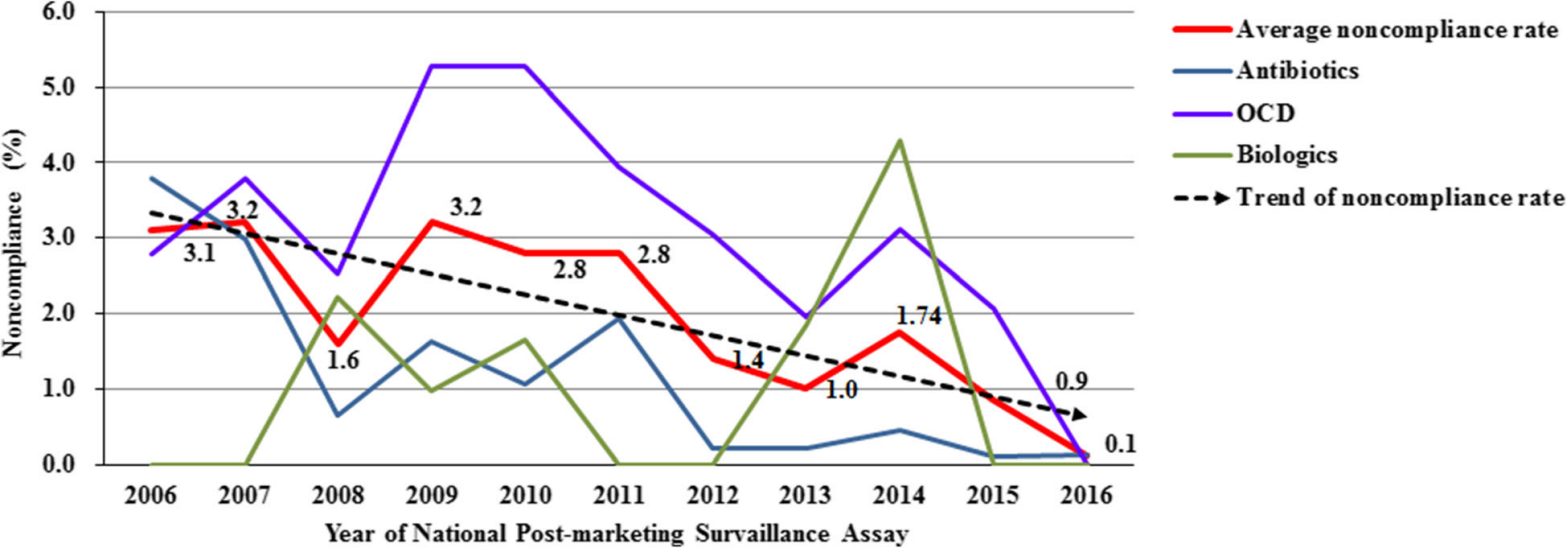
Violation rate of the post-market surveillance assay in veterinary medicines from 2006 to 2016. OCD: other chemical drugs except antibiotics

Testing results for the veterinary medicine samples collected throughout Korea between 2006 and 2016 are shown in Table 2. A total of 18,213 samples, comprising 9504 antibiotics, 8137 OCD, and 572 biologics, were tested, among which a total of 358 samples were found to be noncompliant (average noncompliance rate of 2.0%). The number of noncompliant samples found in different drug groups are antibiotics 103 (1.1%), OCD 248 (3.0%), and biologics 7 (1.2%). The average noncompliance rate by year steadily decreased over the 10 years period, dropping below 1.0% in 2015 and reaching 0.1% in 2016 (Fig. 2).

**Table 2:** Results of national post-market surveillance assay in veterinary medicines from 2006 to 2016

Year	Antibiotics		OCD^a^		Biologics		Total	
	Tested^b^	Noncompliant (%)^c^	Tested^b^	Noncompliant (%)^c^	Tested^b^	Noncompliant (%)^c^	Tested^b^	Noncompliant (%)^c^
2006	606	23 (3.8)	863	24 (2.8)	63	0 (0.0)	1532	47 (3.1)
2007	771	23 (3.0)	687	26 (3.8)	50	0 (0.0)	1508	49 (3.2)
2008	782	5 (0.6)	756	19 (2.5)	45	1 (2.2)	1583	25 (1.6)
2009	916	15 (1.6)	816	43 (5.3)	103	1 (1.0)	1835	59 (3.2)
2010	934	10 (1.1)	663	35 (5.3)	61	1 (1.6)	1658	46 (2.8)
2011	881	17 (1.9)	759	30 (4.0)	55	0 (0.0)	1695	47 (2.8)
2012	918	2 (0.2)	721	22 (3.1)	47	0 (0.0)	1686	24 (1.4)
2013	932	2 (0.2)	664	13 (2.0)	54	1 (1.9)	1650	16 (1.0)
2014	889	4 (0.4)	707	22 (3.1)	70	3 (4.3)	1666	29 (1.7)
2015	1039	1 (0.1)	680	14 (2.1)	24	0 (0.0)	1743	15 (0.9)
2016	836	1 (0.1)	821	0 (0.0)	0	0 (0.0)	1657	1 (0.1)
Total	9504	103 (1.1)	8137	248 (3.0)	572	7 (1.2)	18,213	358 (2.0)

^a^Other chemical drugs except antibiotics (OCD)

^b^The number of tested in the national post-market surveillance assay

^c^The rate of non compliant products, (%) in the national post-market surveillance assay

### Reasons for noncompliance in collected and tested veterinary medicines

Table 3 outlines the causes for noncompliance in veterinary medicines collected and tested between 2006 and 2016. From a total of 358 cases, 283 (79.1%) and 60 (16.8%) cases were deemed noncompliant owing to “insufficiency of major ingredient quantity” and “excess of major ingredient quantity”, respectively. Moreover, 3 (0.8%), 4 (1.1%), 3 (0.8%), and 5 (1.4%) cases were marked as “inadequate pH,” “abnormal characteristics,” “violation of marking standards,” and “others”, respectively.

**Table 3:** The causes of noncompliance in veterinary medicines from 2006 to 2016

Case	Antibiotics	OCD^a^	Biologics	Total (%)^b^
Insufficiency of major ingredient content	92	189	2	283 (79.1)
Excess of major ingredient content	11	49	0	60 (16.8)
Inadequate pH	0	3	0	3 (0.8)
Abnormal characteristics	0	2	2	4 (1.1)
Violation of marking standards	1	2	0	3 (0.8)
Others	1	1	3	5 (1.4)
Total	105	246	7	358

^a^Other chemical drugs except antibiotics (OCD)

^b^the rate of noncompliance%

### Noncompliance rate by active ingredient in collected and tested veterinary medicines

Table 4 shows the noncompliance rates for individual compounds in veterinary medicines collected and tested between 2006 and 2016. For antibiotics, the highest number of noncompliant cases (*n* = 86) were found concerning macrolide drugs, such as tylosin, spiramycin, and erythromycin, followed by 55 cases concerning beta-lactams, such as ampicillin, amoxicillin, and penicillin, and 22 cases concerning tetracyclines. For OCD, the ingredients found to be noncompliant included vitamin A (*n* = 59), probiotics (*n* = 9), and topically applied products such as pet shampoos (*n* = 15), disinfectants (*n* = 8), and glucocorticoids (*n* = 10). For biologics, bovine rota-coronavirus live mixed vaccine, canine parvo and coronavirus live mixed vaccines, porcine transmissible gastroenteritis rotavirus live mixed vaccine, Newcastle live attenuated vaccine, porcine epidemic diarrhoea vaccine, and classic swine fever virus vaccine were found to be noncompliant which is due to their insufficient efficacy. The biological products which couldn’t kill the required number of virus according to the guideline of Animal and Plant Quarantine Agency were listed as non-compliant. Those products also had incorrect pH and may have insufficient concentration of antigens.

**Table 4:** The noncompliant ingredients of products in the post-market surveillance assay from 2006 to 2016

Active ingredients	No. of noncomplience	Noncomplience rate (%)
Vitamin A	59	16.5
Tylosin	48	13.4
Spiramycin	28	7.8
Ampicillin	23	6.4
Amoxicillin	19	5.3
Oxytetracycline	18	5.0
Others	15	4.2
Penicillin	13	3.6
Erythromycin	10	2.8
Gentamicin	9	2.5
Probiotics	8	2.2
Colistin	8	2.2
Dexamethasone	8	2.2
Calcium	5	1.4
Sulfonamides	5	1.4
Chloramphenicol	4	1.1
Doxycycline	4	1.1
Iodine	4	1.1
Ivermectin	4	1.1
Trimethoprim	3	0.8
Cefalexin	3	0.8
Citric acid	3	0.8
Neomycin	3	0.8
Prednisolone	3	0.8
Tiamulin	3	0.8
Amikacin	2	0.6
Apramycin	2	0.6
Benzyl benzoate	2	0.6
Bovine Rota, Corona virus live mixed vaccine	2	0.6
Cefazolin	2	0.6
Cloxacillin	2	0.6
Dicloxacillin	2	0.6
Kanamycin	2	0.6
Melengestrol acetate	2	0.6
Quaternary ammonium salt	2	0.6
Salicylic acid	2	0.6
spectinomycin	2	0.6
Streptomycin	2	0.6
Vitamin B	2	0.6
Amitraz	1	0.3
Newcastle live attenuated vaccine	1	0.3
BPMC	1	0.3
Canine Parvo Corona virus live mixed vaccine	1	0.3
Ceftiofur	1	0.3
Cloprostenol sodium	1	0.3
Enrofloxacin	1	0.3
Florfenicol	1	0.3
Glutaraldehyde	1	0.3
Hydrogen peroxide	1	0.3
Kitasamycin	1	0.3
porcine circo virus vaccine	1	0.3
Phenylbutazone	1	0.3
Porcine transmissible gastroenteritis Rota virus live mixed vaccine	1	0.3
Pyriproxyfen	1	0.3
Tiamphenicol	1	0.3
Trichlorfon	1	0.3
Vitamin E	1	0.3
Porcine Epidemic Diarrhea vaccine	1	0.3
Classic swine fever virus vaccine	1	0.3
Total	358	100

### Seasonal effect on noncompliance in collected and tested veterinary medicines

**Fig. 3:**
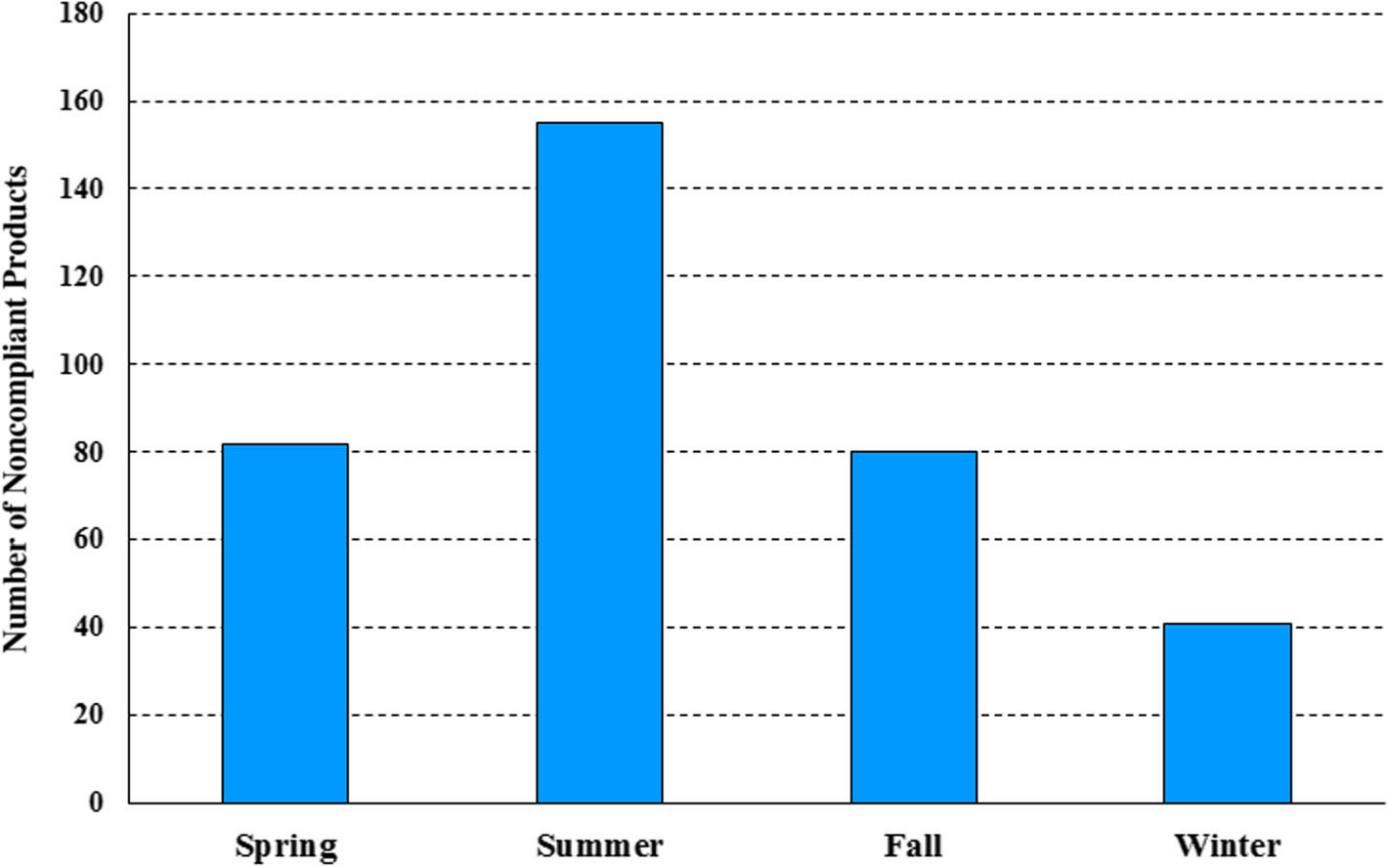
Seasonal effect on noncompliance in collected and tested veterinary medicines

An investigation of noncompliant veterinary medicines collected and tested between 2006 and 2016 showed that out of 358 total cases, the highest number of noncompliant cases (*n* = 155) were found during the summer months (June to August). During spring and fall (March to May and September to November, respectively), the number of noncompliant cases were 82 and 80, respectively, while a relatively lower number of noncompliant cases (41) were found during winter (December to February) (Fig. 3).

## Discussion

Veterinary medicines are essential for the advancement of the livestock industry, and the importance of quality control is emphasizing more due to the increase in the availability and demand of different types of veterinary medicines. Concentration of active ingredient is the most important characteristic of a drug product, as the amount of active ingredient allows to determine the intensities of treatment and toxicity of that particular drug. Moreover, the noncompliance rates of veterinary medicines are also pertaining in the elevation of contamination and drugs residues in the food products of animal origin [14, 15]. The NPMS assessment was initiated in 1964 for the quality control of OCD which are in distribution. Antibiotics and biologics were previously assessed by both PMGTS and NPMS. The control of antibiotics was abolished from PMGTS on 2000–11-07 and the exception biologics which pass ten times consecutively by PMGTS have been incorporated into the NPMS assessment system on 2005–04-09 [3]. So, NPMS is very important for the quality control of veterinary medicines as there is no other assessment system except NPMS.

Testing methods have greatly improved from traditional bioassay examination owing to continued research and development. Improved HPLC and titration methods are now being used for analysis based on the compendia as shown in Table 1. A small surveillance study in Taiwan in the year of 2013 accounted 6 noncompliant cases out of 343 cases (1.7%), which is comparable with our study [16]. The overall violation rate in Korea in the year 2014 was same as the violation rate in Taiwan in 2013. As shown in Fig. 2, noncompliance rates by year showed an annual trend of gradual decrease from 3.2% to 0.1% which indicates that the implication of NPMS in veterinary medicine is helping to improve the quality of them. In particular, antibiotics have shown very dramatic decrease in noncompliance rates (Table 2), illustrating that veterinary medicine manufacturing facilities and quality control standards have significantly improved over time [3, 4]. That such annual trend reflects the effectiveness of the NPMS assessment and KVGMP programs. Meanwhile, no noncompliant cases for OCD were found in 2016, which was due to the testing of a large proportion of disinfectant samples (700 disinfectants out of 821 OCD) compared to the previous years. The disinfectant reinforcement measure due to the 2016 avian influenza outbreak was the behind cause to test many samples only for disinfectants. Vitamin A, which was often found as a noncompliant product in previous years, did not appear in the list of noncompliant products, as we have tested very few samples of Vitamin A among the OCD in 2016.

Of the 358 noncompliant cases, “insufficiency of active ingredient quantity” was the reason for 283 cases, accounting for approximately 79.1% of all noncompliant cases. The next most frequently observed reason was “excess of active ingredient quantity,” with 60 cases observed (approximately 16.8%). In fact, manufacturing facilities cannot always give uniform products. They should have the quality control system and need to check the quality-related parameters batch-wise to comply with the recommended specifications. But, most of the manufacturers do not have quality control system and are unintentionally producing some products which contain less or excess amount of active ingredients. These companies are not also accredited by KVGMP. The second important cause of noncompliance is the using of low quality or potency of active ingredients in veterinary medicine by the manufacturers to reduce the production costs which is investigated by the audit of “Veterinary Management Division” of QIA. So, this report is suggesting to the manufacturers to establish their own quality assurance facilities to produce quality products. Another cause of noncompliance is the storing of the products in improper storage condition particularly in summer season by the sellers and veterinary pharmacies (Fig. 3). It was also recorded in the previous years that the noncompliance rate was increased in summer season due to not maintaining the proper storage condition. This indicates that the seasonal factors, including temperature-related issues during storage and distribution, may act as key factors of noncompliance. Thus, it is suggested to the sellers and distributors to maintain the veterinary medicine in proper storage condition as mention in guidelines.

As shown in Table 4, the noncompliance rate was high among antibiotics with higher molecular weight. The antibiotics were also found to be noncompliant whose active ingredient contain for each dose are high such as tylosin and spiramycin. Among the nutritional supplements, noncompliance was the highest for fat soluble vitamins, such as vitamin A, which has low titre owing to being easily oxidized, as well as a few probiotics, whose cell count drops quickly when preservation conditions are not adequate. Currently, an average of 60–70 cases of biologics both subject to and exempt from national testing are assessed each year, and among these, noncompliance was reported for 2 cases of bovine rota-coronavirus live mixed vaccine, and 1 case each of Newcastle live attenuated vaccine, canine parvo and coronavirus live mixed vaccines, porcine transmissible gastroenteritis rotavirus live mixed vaccine, porcine epidemic diarrhoea vaccine, and classic swine fever virus vaccine.

## Conclusion

Significant improvements in the manufacturing environment and quality control standards have been achieved through the implementation of KVGMP. However, voluntary efforts by manufacturers are still needed to continue the application of KVGMP for the improvement of the quality of veterinary medicines. In particular, OCD showed a high noncompliance rate of about 3%. The Ministry of Agriculture, Food and Rural Affairs (MAFRA) of Korea is expecting to analyse the sales figures from the Korea Animal Health Products Association, and the NPMS assay results from QIA accordingly, to increase the statistical feasibility of the NPMS assessment results. The MAFRA is also expecting to increase the number of test samples for OCD, as they present relatively high noncompliance rates. It is also expected by MAFRA to reinforce testing during the vulnerable summer season. Proactive self-quality control by manufacturers for improved veterinary medicine quality must be promoted; while simultaneously, the efforts to establish an effective quality control system must continue as well.

## Abbreviations

HPLC: High performance liquid chromatography.
KVGMP: Korea veterinary good manufacturing practice
MAFRA: Ministry of agriculture, food, and rural affairs
NPMS: National post-market surveillance
OCD: Other chemical drugs except antibiotics
PMGTS: Pre-market government testing system
QIA: Animal and plant quarantine agency
